# "Neck Dyskinesia With Olanzapine-a case report"

**Published:** 2004

**Authors:** Ajeet Sidana, Gurvinder Pal Singh, Rakash Pal Sharma

**Affiliations:** * Senior Lecturer, Deptt. of Psychiatry, Govt. Medical College and Hospital, Chandigarh - 160047; ** Senior Resident, Deptt. of Psychiatry, Govt. Medical College and Hospital, Chandigarh - 160047; *** Senior Resident, Deptt. of Psychiatry, Govt. Medical College and Hospital, Chandigarh - 160047


### "Neck Dyskinesia with Olanzapine-a case report"

Sir,

Tardive dyskinesia (TD) is a serious side effect with traditional antipsychotics. Olanzapine is an atypical antipsychotic agent with a reported lack of propensity to cause T.D. Recently, few reports of olanzapine induced tardive dystonia and dyskinesia are reported (Tollefson et al, 1997, Ananth & Kenan, 1999 and Dunayeich & Srakowski, 1999). Even though some reports indicate that olanzapine can improve T.D. (Almedia, 1998, Littrell et al, 1998 and Brien et al, 1998), here authors reported a rare case of neck dyskinesia in a paranoid schizophrenic male who was on olanzapine since last 7 months.

### Case Report

Mr. S. a 43 yrs. old Hindu male working in insurance company, visited psychiatry OPD in Nov. 2002 and was diagnosed as paranoid schizophrenia. He had no past history of any psychiatric/medical disorder or any treatment with traditional antipsychotics. Patient was put on olanzapine 5mg/day initially and gradually dose was builtup to 15mg/day and patient was stabilized on the same dose.

Patient reported significant improvement in symptomatology in next 2-3 months. In May, 2003, abnormal neck movements were reported by the spouse.

On examination of neck, there were involuntary, irregular, choreoathetoid movements of posterior triangle of neck. Contractions were evident in both right and left cervical portion of trapezius muscle (nodding of neck) with marked distress and sensation that something is climbing over his occipital part of scalp. AIMS score was 12. There were no signs of other EPS. All routine and special investigations (CBP, RFTs, LFTs, TFTs) were undertaken to rule out other causes before arriving at final conclusion of olanzapine induced dyskinetic movement. The dose of olanzapine was reduced to 10mg/day, but no improvement was observed. In June 2003 tetrabenazine was added with olanzapine 10 mg/day for next 2 months but again there was no improvement in abnormal movement. Psychotic features of this patient remitted completely. Olanzapine was stopped in July,2003 and tetrabenazine 25mg/ day was continued. During the next 3 months dyskinetic movement disappeared completely.

### Discussion:

Eventhough claims of low risk of TD with olanzapine and even improvement in TD with olanzapine therapy (Almedia, 1998, Littrell et al, 1998 and Brien et al, 1998). In this case, Olanzapine was the only possibility for the neck dyskinesia. The early onset of TD in young drug naïve patient requires the vigilant attitude of clinician. Now a days the increasing use of olanzapine in behavioural disorder in the absence of psychosis, puts the patient on unnecessary risk of TD. Our finding suggests that drug naïve patients are equally at risk for TD and one should always look for the TD and other movement disorder even in the early part of treatment. More research is required to settle this issue.

### REFERENCES

Almedia OP. (1998) Olanzapine for the treatment of tardive dyskinesia (letter). Journal of Clinical Psychiatry, 59:380-381.

Ananth J, Kenan J. (1999) Tardive dyskinesia associated with Olanzapine monotherapy (letter). Journal of Clinical Psychiatry, 60,870.

Dunayevich E. Strakowski S.M. (1999) Olanzapine induced tardive dyskinesia (letter).American Journal of Psychiatry, 156:1662.

Littrell KH, Johnson CG, Littrell S, et al. (1998) Marked reduction of tardive dyskinesia with olanzapine. Archives General of Psychiatry, 55:279-280.

O' Brien J, Barber R. (1998) Marked improvement in tardive dyskinesia following treatment with olanzapine in an elderly subject (letter). British Journal of Psychiatry, 172:186.

Tollefson GD, Beasley CM Jr., Tamura RN, et al. (1997) Double-blind controlled long term study of the comparative incidence of treatment emergent tardive dyskinesia with olanzapine or haloperidol. American Journal of Psychiatry, 154:1248-1254.

**Ajeet Sidana***, Senior Lecturer, **Gurvinder Pal Singh ****, Senior Resident,**Rakash Pal Sharma ***** Senior Resident, Deptt. of Psychiatry, Govt. Medical College and Hospital, Chandigarh - 160047
*Correspondence

